# Correspondence

**Published:** 1882-03

**Authors:** E. S. Dunster

**Affiliations:** Prof. Obs’ts and D’s Women and Children, University of Michigan


### CORRESPONDENCE.

### LARGE DOSES OF CHLORAL.

UNIVERSITY OF MICHIGAN, FEBRUARY 6, 1882.

*Editor Independent Practitioner:*

DEAR SIR.—Referring to the case reported by Dr. Griswold in the current number of the INDEPENDENT PRACTITIONER, of recovery after taking 280 grains of chloral, I find in my *Index Rerum* the following cases: *Phila. Med. Times*, Oct. 15, 1870, 460 grs.; *Ibidem*, March 22, '73, 260 grs; *Dublin Jour. Med. Science*, Aug., 1874, "Over 1 ounce;" *Monthly Abstract Med. Sciences*, June, 1876, "over 400 grs."

Very respectfully,

Your Obedient Servant,

E. S. DUNSTER, M. D.,

*Prof. Obs'ts and D's Women and Children, University Mich.*

